# Combined morphological and phylogenomic re-examination of malawimonads, a critical taxon for inferring the evolutionary history of eukaryotes

**DOI:** 10.1098/rsos.171707

**Published:** 2018-04-04

**Authors:** Aaron A. Heiss, Martin Kolisko, Fleming Ekelund, Matthew W. Brown, Andrew J. Roger, Alastair G. B. Simpson

**Affiliations:** 1 Department of Invertebrate Zoology and Sackler Institute for Comparative Genomics, American Museum of Natural History, New York, NY 10024, USA; 2 Centre for Comparative Genomics and Evolutionary Bioinformatics, Department of Biology, Dalhousie University, Halifax, Nova Scotia B3H 4R2, Canada; 3 Centre for Comparative Genomics and Evolutionary Bioinformatics, Department of Biochemistry and Molecular Biology, Dalhousie University, Halifax, Nova Scotia B3H 4R2, Canada; 4 Institute of Parasitology, Biology Centre, Czech Academy of Sciences, Branišovská 31, 370 05 České Budějovice, Czech Republic; 5 Department of Biology, University of Copenhagen, Universitetsparken 15, 2100 Copenhagen, Denmark; 6 Department of Biological Sciences, Mississippi State University, Starkville, MS 39762, USA

**Keywords:** eukaryote evolution, protist, Excavata, Metamonada, transcriptomics, ultrastructure

## Abstract

Modern syntheses of eukaryote diversity assign almost all taxa to one of three groups: Amorphea, Diaphoretickes and Excavata (comprising Discoba and Metamonada). The most glaring exception is Malawimonadidae, a group of small heterotrophic flagellates that resemble Excavata by morphology, but branch with Amorphea in most phylogenomic analyses. However, just one malawimonad, *Malawimonas jakobiformis*, has been studied with both morphological and molecular-phylogenetic approaches, raising the spectre of interpretation errors and phylogenetic artefacts from low taxon sampling. We report a morphological and phylogenomic study of a new deep-branching malawimonad, *Gefionella okellyi* n. gen. n. sp. Electron microscopy revealed all canonical features of ‘typical excavates’, including flagellar vanes (as an opposed pair, unlike *M. jakobiformis* but like many metamonads) and a composite fibre. Initial phylogenomic analyses grouped malawimonads with the Amorphea-related orphan lineage *Collodictyon*, separate from a Metamonada+Discoba clade. However, support for this topology weakened when more sophisticated evolutionary models were used, and/or fast-evolving sites and long-branching taxa (FS/LB) were excluded. Analyses of ‘–FS/LB’ datasets instead suggested a relationship between malawimonads and metamonads. The ‘malawimonad+metamonad signal’ in morphological and molecular data argues against a strict Metamonada+Discoba clade (i.e. the predominant concept of Excavata). A Metamonad+Discoba clade should therefore not be assumed when inferring deep-level evolutionary history in eukaryotes.

## 1. Introduction

Most current views of the diversity of eukaryote life divide almost all known taxa into three massive assemblages [1–5]. These are: (i) Amorphea, which includes animals, fungi, choanoflagellates, many amoebae and most slime moulds; (ii) Diaphoretickes, encompassing land plants, almost all algae, and many heterotrophs like ciliates and foraminifera; and (iii) Excavata, which includes the euglenid algae, diverse parasites (e.g. trypanosomatids, trichomonads, *Giardia*), and various free-living protozoa like jakobids, heteroloboseids and *Carpediemonas* (alternative names for similar major assemblages are sometimes used [2]). The Excavata grouping contains two main subclades, Metamonada and Discoba, which are each robustly supported by molecular phylogenetics [6,7]. Some taxa in both Metamonada and Discoba are so-called ‘typical excavates’, organisms that share a characteristic suspension-feeding groove supported by a complex and specific flagellar apparatus cytoskeleton, as well as a vane-bearing posterior flagellum. These features unite Excavata morphologically [8].

Despite this eukaryote-wide phylogenetic framework, there remain a number of enigmatic protist lineages with poorly resolved evolutionary affinities. The most extraordinary example is Malawimonadidae. Malawimonads are small aerobic heterotrophic flagellates with a feeding groove [9]. An electron microscopy study of *Malawimonas jakobiformis* identified most of the ‘typical excavate’ cytoskeletal features [8–10], and phylogenies of one or a few slowly-evolving marker genes usually place malawimonads as a relative of some or all other excavates, though usually with only modest support [11–14]. By contrast, most phylogenomic analyses, which examine scores-to-hundreds of genes, show malawimonads branching separately from other excavates, and instead place them with Amorphea [7,15–22]. If accurate, this inference profoundly impacts our understanding of the history of eukaryotic cells. Assuming the ‘excavate-type’ cell architecture is truly homologous in malawimonads and other ‘typical excavates’, it implies that the last common ancestor of all living eukaryotes was a ‘typical excavate’ itself, under the most popular model for the placement of the root of the tree of eukaryotes [21,23]. This is a remarkably specific inference about a pivotal species that lived more than a billion years ago.

To date, only one species of malawimonad has been described, *Malawimonas jakobiformis*. All published morphological information is from one strain of *M. jakobiformis* [9], while almost all analyses of molecular sequences employ data from two strains, the type strain of *M. jakobiformis* and a second, undescribed strain usually known informally as ‘*Malawimonas californiana*’ [7,15,16,21,24–26]. Given the importance of malawimonads for understanding the deep-level evolutionary history of eukaryotes, this is a perilously narrow base of information.

Recently, the mitochondrial genome was reported from a third malawimonad, ‘strain 249’ [27]. Here, we describe strain 249 as *Gefionella okellyi* n. gen. n. sp. (see Taxonomic summary, below). *Gefionella okellyi* proves to be sister to the previously studied malawimonads. We determined the three-dimensional architecture of its flagellar apparatus cytoskeleton, and conducted phylogenomic analyses incorporating transcriptomic data. Our new data provide a broader base of understanding for malawimonads, allowing for a critical examination of the affinities of this mysterious group.

## 2. Material and methods

### 2.1. Collection, isolation and culturing

*Gefionella okellyi* strain 249 was isolated from agricultural soil from Foulum, Jutland, Denmark [56^o^29′47.8^″^ N 9^o^34′32.2^″^ E]. Monoeukaryotic cultures were maintained at 21°C in tilted, sealed 15-ml tubes containing 3 ml of 25%-strength cerophyl medium (ATCC medium 802; ScholAR Chemistry, West Henrietta, NY, USA), with mixed unidentified bacteria. Bulk cultures were grown in 4-l flasks containing 1.0–1.5 l of 100% cerophyl medium, on a rotary shaker (120 rpm), at room temperature (RT).

### 2.2. Microscopy

Live cultures were observed using phase contrast and differential interference contrast optics, with 100× oil-immersion objectives and a 1.6× ‘optovar’ lens, and documented using a 1.4-megapixel camera.

Cells were fixed for scanning electron microscopy (SEM) using an osmium tetroxide vapour protocol [28], collected on 2.0-µm Isopore filters (Millipore), dehydrated through an ethanol series, critical-point dried in CO_2_, and sputter-coated with gold/palladium. Cells were imaged using only the secondary electron detector of the SEM at 20 keV.

For transmission electron microscopy (TEM), 3 ml of culture was concentrated by centrifugation (3000 × *g* for 5 min), fixed in 2.5% glutaraldehyde, rinsed twice, postfixed in 1% osmium tetroxide, and rinsed three times. All steps through the first post-OsO_4_ rinse were performed in 50% cerophyl medium; the final two rinses were in distilled water. Cells were enrobed in 2% agarose, dehydrated through an ethanol series (30–50–70–80–90–95 × 2–100 × 3, 10 min each change), then propylene oxide (50% with ethanol, then three changes in pure reagent), and embedded in SPI-Pon resin (SPI) with intermediate 1 : 2 and 2 : 1 changes in resin : propylene oxide. Approximately 50-nm-thick serial sections were cut with a diamond knife, mounted on pioloform film in slot grids, stained with uranyl acetate (10 min) and lead citrate (5 min), and observed on a TEM equipped with a goniometer stage and a 14-megapixel camera. Eighteen series of 8–21 sections were documented, plus several shorter series. A three-dimensional (3-D) model was derived from one 21-section series, as described previously [29]. Briefly, the micrographs were first annotated by hand in a vector drawing program. The vector data were then imported to a 3-D modelling program, where they were aligned and scaled appropriately. Annotations corresponding to the same structure (e.g. the same microtubule) were identified, and model structures were constructed using the annotations as a framework. The final model included significant preparation artefacts (e.g. compression, skew), which were corrected by hand. All stages of the reconstruction process occurred with reference to multiple other series; no structure was represented in the model unless it could be identified in at least one other series.

### 2.3. Transcriptomics and phylogenetics

Approx. 6.6 × 10^9^ cells (in 3 l media) were concentrated by centrifugation (2000 × *g* for 10 min) and resuspended in 100 ml TRIzol (Ambion), from which RNA was isolated according to the manufacturer's instructions. Purified RNA (550 ng) was submitted for library construction and Illumina sequencing (5.9 × 10^7^ 101 bp paired-end reads; Macrogen, Seoul, South Korea).

Raw data were assembled into contigs using ‘Inchworm’ from the ‘Trinity’ package [30]. Low-*k*-mer contigs were removed to exclude mild contamination introduced during sequencing. Sequences were added to a published 159-gene phylogenomic dataset using an in-house Python pipeline [31,32]. This dataset also included recently reported transcriptome data from shorter-branching metamonads, including *Trimastix marina* [33]. All phylogenetic trees based on single-gene datasets were inspected by eye, and paralogues and potential lateral transfers were removed. Additionally, all bipartitions in single-gene trees with bootstrap proportions (BP) greater than 70% were cross-checked against a reference tree of eukaryotes, and conflicting bipartitions were examined by eye. The final dataset as analysed here had 84 taxa and 42 564 sites, with *G. okellyi* showing 75% site coverage.

The dataset was initially analysed using maximum likelihood (ML) as implemented by RAxML v. 7.8.1 [34] with the site-homogeneous evolutionary model LG+Γ+I. Parameters were estimated by the software and 500 bootstrap replicates were performed. A second ML analysis was conducted in IQ-TREE v. 1.5.5 [35] using a site-heterogeneous model (LG+C60+F+Γ4), with robustness assessed via ‘ultrafast’ bootstrap approximation (1000 replicates). We also performed a Bayesian analysis using PhyloBayes-MPI [36] on the full-taxon 159-gene dataset, using the site-heterogeneous CAT-GTR+Γ4 model, with four chains sampled every second generation for 24 000 generations. This computation- and time-expensive analysis still showed only two chains converging (maxdiff = 0.168), which were assessed after discarding the first 20% of generations as burn-in.

The impact of fast-evolving sites and long-branching taxa (FS/LB) on the phylogenetic inference was assessed in ‘–FS/LB’ analyses as follows. Taxa were sorted by branch length (as inferred under ML using the LG+Γ+I model), and 35 of them (42%) were sequentially removed, to generate 36 taxon sets (including the original alignment). To eliminate the issue of the unknown position of the root, we calculated all pairwise branch lengths and used the average of the ten longest tip-to-tip distances for each taxon as the branch length metric. Fast evolving sites were then removed for each of the 36 taxon sets as follows. An evolutionary rate was estimated for each site using Dist_Est [37], then sites were sorted from fastest- to slowest-evolving and removed by thousands until 30 000 sites were excluded (generating 30 alternative datasets for each original one). Each of the 1080 datasets (36 × 30) was then bootstrapped using 100 rapid bootstraps in RAxML (model setting PROTCATLGF). Finally, the dataset with 23 000 fast-evolving sites and 22 long-branch taxa removed was selected for detailed phylogenetic analysis as above, including (i) a maximum-likelihood analysis using the LG+C60+F+Γ4 model in IQ-TREE, with a 1000-replicate ‘ultrafast’ bootstrap analysis, (ii) a maximum-likelihood analysis under the LG+Γ4+I model with 500 bootstrap replicates in RAxML (v. 8.1.16), and (iii) a Bayesian analysis using PhyloBayes-MPI under the CAT-GTR+Γ4 model, as described above, but with convergence among all four chains observed after 32 000 generations (maxdiff = 0.063).

## 3. Results

### 3.1. Morphology

**Figure 1.**
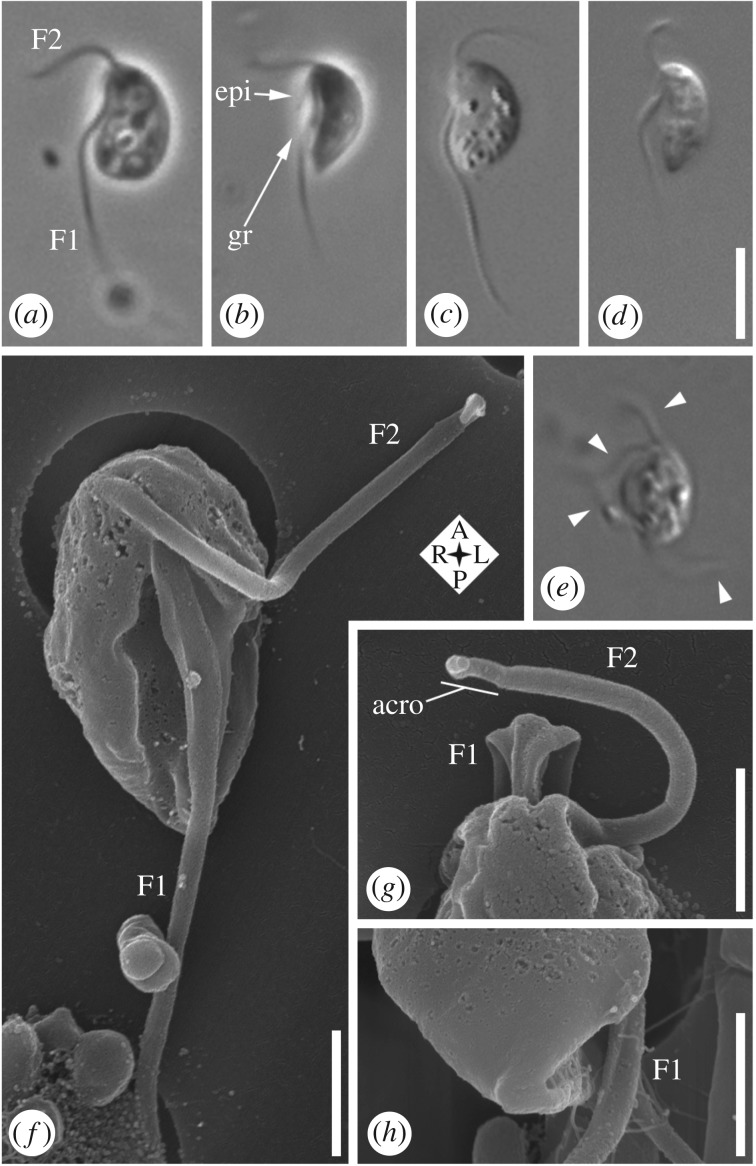
Light microscopy and scanning electron microscopy (SEM) of *Gefionella okellyi* n. gen. n. sp. (*a*–*e*) Phase contrast (*a*,*b*) and differential interference contrast (*c*–*e*) images of live cells, with (*e*) showing dividing cell with four flagella in two pairs (arrowheads). (*f*) SEM, showing ventral view, including feeding groove, and vanes on posterior flagellum (F1). (*g*) SEM of anterior end of cell from dorsal side; note origins of the pair of vanes on F1 (distal to origin of vanes, flagellum rotates clockwise with respect to cell). (*h*) SEM of posterior end of cell, showing termination of vanes on F1, and robust nature of right margin of posterior end of feeding groove (in foreground). acro, acroneme; epi, epipodium; F1, posterior flagellum; F2, anterior flagellum; gr, groove. Scale bars: (*a*–*e*, in *d*) 5 µm. (*f*–*h*) 1 µm.

**Figure 2.**
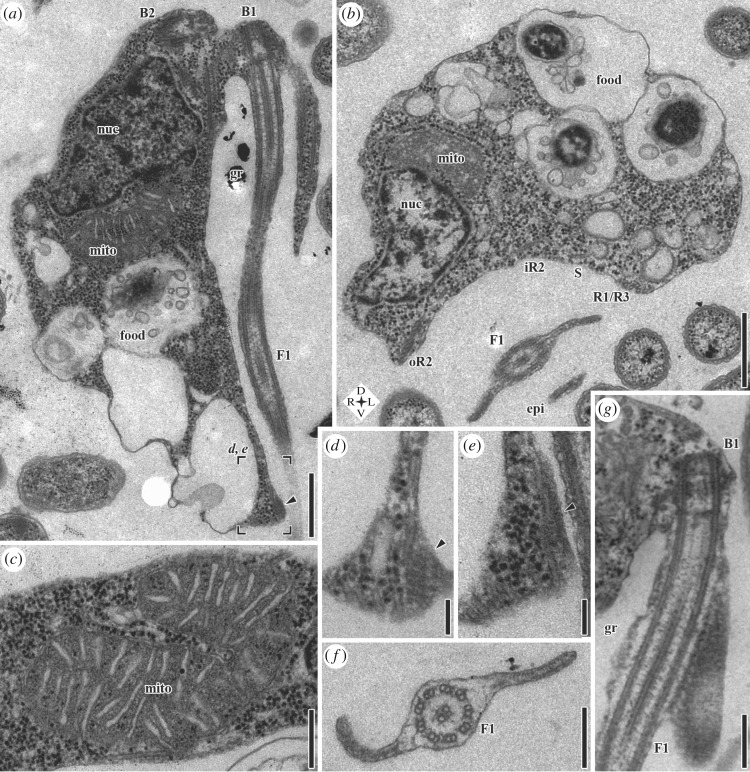
Transmission electron microscopy (TEM) of *G. okellyi*. (*a*) Longitudinal section; note marks for position of images *d* and *e*, detailing composite fibre (arrowhead). (*b*) Transverse section, showing architecture of ventral feeding groove; note compass rose for orientation. (*c*) Mitochondrion, showing discoidal cristae in transverse section. (*d*,*e*) Serial sections of posterior part of right margin of groove in same cell as *a*, showing striated and dense portions of composite fibre (arrowhead), respectively. (*f*) Transverse section of posterior flagellum (F1), showing vanes. (*g*) Longitudinal section of proximal portion of posterior flagellum, showing origin of dorsal vane, and striations of its lamella. B1, basal body 1 (of posterior flagellum); B2, basal body 2 (of anterior flagellum); epi, epipodium; F1, posterior flagellum; food, food vacuole; gr, groove; iR2, inner portion of microtubular root 2; mito, mitochondrion; nuc, nucleus; oR2, outer portion of microtubular root 2; R1, microtubular root 1; R3, microtubular root 3; S, singlet microtubular root. Scale bars: (*a*) 500 nm, (*b*) 500 nm, (*c*) 250 nm, (*d*) 100 nm, (*e*) 100 nm, (*f*) 200 nm, (*g*) 250 nm.

Live interphase cells have an approximately 6 µm long main cell body (4.4–7.2 µm; av. 5.9; s.d.: 0.6; *n* = 30). The main cell body is generally bean-shaped (though sometimes with a pointed posterior, which may generate a temporary extension up to 1.5 µm) and has a ventral feeding groove (figure 1*a*–*d*). One groove margin may project slightly as an ‘epipodium’ (see below; figure 1*b*). Cells have two subapical flagella (figure 1*a*–*d*) unless dividing, when four can be present in two pairs (figure 1*e*; arrowheads). The anterior flagellum (F2) originates to the right of the posterior flagellum (F1: figure 1*f*,*g*; note that 1*f* is a ventral view—the cell's right is to the left of the image). F2 is about the length of the cell (figure 1*a*–*d*), has an acronematic tip (i.e. narrows abruptly: figure 1*g*), and sweeps from ventral to anterodorsal (figure 1*a*–*d*). The posterior flagellum (F1) is usually 2–2.5 times cell length, and runs adjacent to or within the groove, then trails posteriorly (figure 1*a*–*d*). Its proximal ∼third bears two vanes, on opposite sides (visible by SEM; figure 1*f*,*g*). The right margin of the posterior end of the groove is quite robust (figure 1*h*), reflecting the presence of the composite fibre (see below). Cells feed on individual bacteria (figure 2*a*,*b*), and can form small rounded cysts (not shown). Cells excyst readily in culture upon addition of fresh media. In agreement with its isolation from soil, the strain grows in freshwater media (see Material and methods); its tolerance for salt, as well as for pH and other environmental variables, was not tested here.

**Figure 3.**
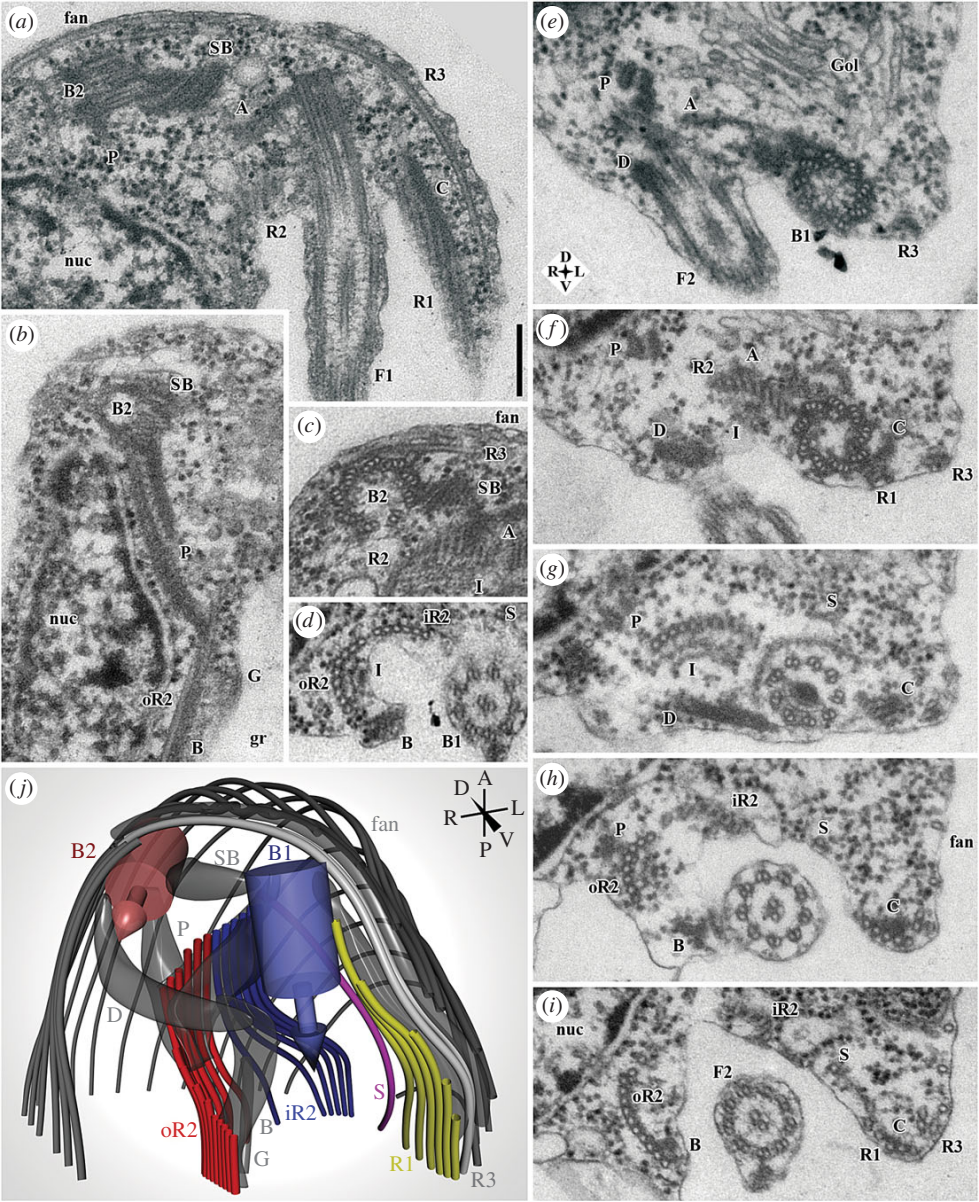
Flagellar apparatus of *G. okellyi*, represented by transmission electron microscopy (TEM) and 3-D modelling. (*a*) Anterior end of cell, showing flagellar apparatus, including basal body 2 (B2) and posterior flagellum (F1), with basal body 1 forming the latter's base. (*b*) Detail of ‘P’ fibre, and section of inconspicuous ‘G’ fibre. (*c*) Transverse section through basal body 2 (B2), showing origin of microtubular root 3 (R3) and part of dorsal fan. (*d*) Detail of ‘I’ and ‘B’ fibres associated with microtubular root 2 (seen here just posterior to split into iR2 and oR2); note striations of ‘B’ fibre. (*e*–*i*) Series of sections through anterior portion of cell, showing origins and organization of microtubular elements supporting ventral groove (and dense fibre ‘D’ connecting the basal bodies); note compass rose for orientation. Origins of microtubular roots 1 and 2 (R1, R2) are shown in *f*, origin of singlet root S in *g*, division of R2 into iR2 and oR2 in *h*, origin of ‘B’ fibre in *h*, and connection of ‘B’ to oR2 in *i*. (*j*) Model of proximal portion of flagellar apparatus, rendered from a 21-section series; note compass for orientation. A, ‘A’ fibre; B, ‘B’ fibre; B1, basal body 1 (of posterior flagellum); B2, basal body 2 (of anterior flagellum); C, ‘C’ fibre; D, dense fibre; F1, posterior flagellum; F2, anterior flagellum; fan, dorsal fan of microtubules; G, ‘G’ fibre; Gol, Golgi apparatus; gr, groove; I, ‘I’ fibre; iR2, inner portion of microtubular root 2; nuc, nucleus; oR2, outer portion of microtubular root 2; P, ‘P’ fibre; R1–R3, microtubular roots 1–3; S, singlet microtubular root; SB, striated band. Scale bar: (*a*–*i*; in *a*) 200 nm.

The nucleus lies anteriorly, in the cells' right side (figure 2*a*,*b*; in 2*b* the cell's right is to the left of the image; see also electronic supplementary material, figure S1*e*–*g*). There are typically 3–4 mitochondria. One or more are associated with the nucleus (figure 2*a*,*b*) and positioned toward the centre of the cell with respect to the nucleus. The remaining mitochondria are spread throughout the cell (see electronic supplementary material, figure S3*a*). The mitochondria have discoidal cristae (i.e. flattened, but with narrow inner membrane connections that are rarely sectioned; figure 2*c*). The Golgi apparatus lies near the basal bodies (figure 3*e*). Food vacuoles are mostly located posteriorly (figure 2*a*). The right margin of the posterior end of the groove contains a small ‘composite fibre’ (figure 2*a*, arrowhead), with striated and dense components (figure 2*d* and *e* respectively). Each vane on the posterior flagellum (F1) is supported by a lamella (figure 2*f*) that has fine striations visible in grazing sections (figure 2*g*; electronic supplementary material, figure S1*a*).

The proximal flagellar apparatus is shown in figure 3, with a 3-D model in figure 3*j* (see also electronic supplementary material, figures S1–S4). The basal bodies are approximately 300 nm long, with a very thin cartwheel and a simple transitional plate (figure 2*g*; see also figure 3*e*–*g*). The basal bodies lie at right angles, separated by 200–300 nm (figures 2*a* and 3*a*). A striated band (SB) connects the left side of the anterior basal body (B2) to the proximal end of the posterior basal body (B1; figure 3*a*–*c*,*e*–*g*). A dense distal fibre (D; figure 3*e*–*g*) connects their distal ends.

Microtubular root R3 originates alongside B2. It consists of two microtubules associated with a thin non-microtubular fibre, and extends down the left side of the cell (figure 3*a*,*c*,*e*–*i*). A ‘dorsal fan’ of spaced sub-membrane microtubules originates along R3 and the anterior side of B2 (figure 3*c*).

Three microtubular roots, ‘R1’, ‘R2’ and ‘S’, originate near B1, and are associated with the ‘typical excavate’ set of non-microtubular fibres: ‘A’, ‘B’, ‘C’ and ‘I’ (see [8]). R1, eventually with six microtubules, originates on the left side of B1, and has the narrow, dense C fibre on its dorsal side (figure 3*a*,*f*–*i*). R2 originates on the right side of B1 as a curved row of about eight microtubules, connected to B1 on its dorsal side by the narrow A fibre (figure 3*a*,*c*,*e*,*f*). The I fibre adheres to the ventral face of R2, and is thick (approx. 75 nm), with a complex laminate structure (figure 3*c*,*d*,*g*; electronic supplementary material, figure S1*b*–*d*). The B fibre is narrow and striated. It originates near B1 (and one end of the distal fibre: see above), and heads right to associate with the right edge of R2 (figure 3*d*,*h*). Root ‘S’ is a single microtubule that originates near the dorsal side of R2 (figure 2*f*–*i*; see electronic supplementary material, figure S1).

A novel structure, the P (=‘paired’) fibre, consists of two electron-dense and striated elements joined by fine material. It runs alongside the nucleus and connects the dorsal/right face of R2 to the posterior side of B2 (figure 3*a*,*b*,*e*–*h*).

Soon after its origin, R2 splits into an inner ‘iR2’ with six microtubules and an outer ‘oR2’ that grows to 15+ microtubules by addition along its outer (right) edge. The I fibre continues with oR2 only. The I fibre ends approximately 400 nm after the split, distal to which the B fibre connects to the ventral/rightmost part of oR2 (figure 3*i*), and the P fibre ends against the dorsal side of oR2 (figure 3*h*). As iR2 and oR2 diverge, a narrow ‘G’ (=‘groove’) fibre originates against the ventral face of iR2, but bridges the gap between iR2 and oR2, and continues posteriorly with oR2 (electronic supplementary material, figures S1*d*–*g* and S2*k*). Several individual microtubules diverge from both oR2 and iR2 to support the groove membrane between them (electronic supplementary material, figures S1*g* and S2*k*,*l*), while S joins R1, and R1 frays into individual microtubules (electronic supplementary material, figure S2*h*,*i*).

More posteriorly the microtubules derived from R1, R2, R3 and S arrange into three elements. (i) The oR2 (see above) supports the right wall of the groove but is reduced to about three microtubules by the posterior end, where it associates with the composite fibre (figure 2*d*,*e*; electronic supplementary material, figure S3). (ii) The R3 and three R1 microtubules support the epipodium, terminating at its end (electronic supplementary material, figure S2*c*–*f*). (iii) A group consisting of three microtubules from R1, the singlet S, and about three microtubules of iR2 defines the left edge of the groove posterior to the epipodium (electronic supplementary material, figure S2*d*,*e*), and converges at the cell's posterior with the remains of oR2 and the composite fibre (electronic supplementary material, figure S3*e*–*h*).

### 3.2. Phylogenomics

**Figure 4.**
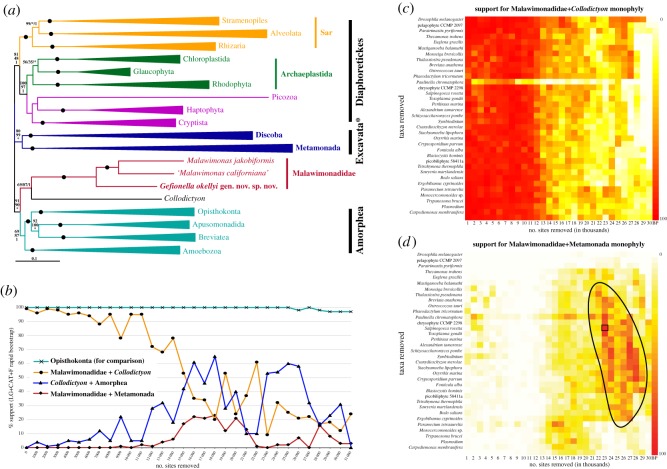
(*a*) Phylogenetic tree of eukaryotes, based on 159 genes, with all sites and 84 taxa included. Maximum-likelihood (ML) tree shown was inferred under LG+C60+Γ4+F model of sequence evolution using IQ-TREE. For clades summarized as triangles, length shows average total branch length. ‘Excavata’ is labelled with an asterisk to signify that this clade does not include the ‘excavate taxon’ Malawimonadidae, and to also flag the contested nature of this clade (compare with figure 5). Statistical support values are, in order: LG+C60+Γ4+F model ‘ultrafast’ bootstrap approximation (UFboot) from IQ-TREE, LG+Γ4+F model bootstrap support (BP) from RAxML, and CAT-GTR+Γ4 model Bayesian posterior probabilities (from the two converged chains) in PhyloBayes-MPI. Filled circles represent maximal support (i.e. 100/100/1.0). Unlabelled branches received less than 50% UFboot support. Asterisks denote branches that were not recovered in inferred phylogeny for a given analysis. The full phylogenetic tree is shown in the electronic supplementary material, figure S5. (*b*) Chart depicting ML BP for groups on intervals along removal of fast-evolving sites. *Y*-axis denotes BP and *X*-axis denotes number of fastest-evolving sites removed. Support for Opisthokonta serves as an indicator of whether sufficient data is left for deep-level phylogenetic inference; other taxa summarized as triangles in *a* were similarly supported (data not shown). (*c*,*d*) Heat maps showing BP for a Malawimonadidae+*Collodictyon* clade (*c*) or a Malawimonadidae+Metamonada clade (*d*) with respect to removal of fast-evolving sites (*X*-axis, in thousands) and fast-evolving taxa (*Y*-axis). Pure white denotes 0% BP and pure red denotes 100% BP (rightmost columns demonstrate colour scale). Black box in *d* identifies dataset selected for full analysis shown in figure 5. Anomalous support values observed with removal of *Paulinella chromatophora* are probably result of unbalanced taxon sampling within Sar, which has an effect on overall tree topology. All analyses in *b*–*d* used LG+CAT+F model and rapid bootstrapping in RAxML.

Our phylogenomic analyses demonstrated that *Gefionella* is the deepest branch in the malawimonad clade, with maximum support (figure 4*a*; electronic supplementary material, figure S5). In the full-dataset phylogenomic tree, malawimonads were sister to *Collodictyon,* with variable support (LG+C60+Γ4+F ML ultrafast bootstrap approximation in IQ-TREE (UFboot)—69%; LG+Γ4+F ML bootstrap percentage (BP)—87%; CAT-GTR Bayesian posterior probability (PP)—1.0). This group branched in a clan with Amorphea (i.e. opisthokonts, apusomonads, breviates and Amoebozoa) with quite strong support (UFboot 91%; BP 90%; PP 1.0), separately from Metamonada and Discoba. The ML analyses grouped metamonads and discobids as a clade (‘Excavata*’ in figure 4*a*; LG+Γ4+F BP 99%, but LG+C60+Γ4+F UFboot 80%), which was placed on the unrooted tree between the malawimonads+*Collodictyon*+Amorphea clan and an unsupported Diaphoretickes clan. The CAT-GTR PhyloBayes analysis instead placed metamonads and discobids as two separate clades between Diaphoretickes and the malawimonads+*Collodictyon*+Amorphea clan (with PP of 1.0 for all separating bipartitions). Thus, the positions of both malawimonads and metamonads were incompletely resolved in these initial analyses, though all of them separated malawimonads from (other) excavates.

Ancient phylogenetic signals are expected to be largely erased from rapidly evolving sites due to multiple substitutions; conversely, what appear to be well-supported deep phylogenetic relationships may be artefacts that depend on the fastest evolving sites being included [32,38–40]. We performed fast-site-removal analyses, in which sites with the highest estimated evolutionary rates were successively removed, and statistical support for important clades was tracked (figure 4*b*). This fast-site removal was also combined with the successive exclusion of the longest-branching taxa (‘–FS/LB’ analyses; figure 4*c*,*d*). Rapid bootstrap support (computed in RAxML) for the Malawimonadidae+*Collodictyon* clade declined with removal of fast evolving sites, falling from 100% to approximately 50% after removal of 15 000 sites, and later to approximately 25% (figure 4*b*,*c*). Support for Metamonada branching with Discoba showed a similar pattern of decline (electronic supplementary material, figure S6). By contrast, support for Opisthokonta (plotted as a control clade in figure 4*b*) remained at or near 100% throughout this site removal series. In fact, most other major eukaryote groups (e.g. those depicted as triangles in figure 4*a*) still received (near-) maximal support after removal of 15 000 sites (data not shown), further indicating that the collapse of support for Malawimonadidae+*Collodictyon* and Metamonada+Discoba was not due to a general loss of deep phylogenetic signal.

**Figure 5.**
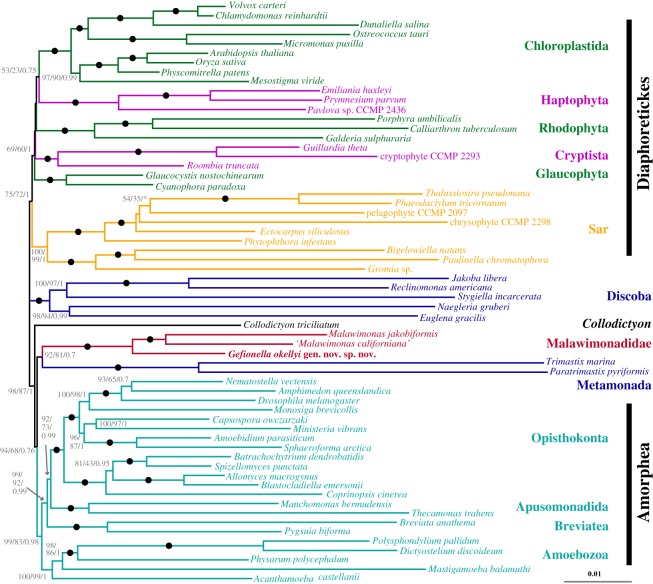
Phylogenetic tree of eukaryotes, based on 159 genes, with 23 000 fast-evolving sites and 22 long-branch taxa excluded from initial dataset. Tree shown was inferred under LG+C60+Γ4+F model of sequence evolution using IQ-TREE. Statistical support values are, in order: LG+C60+Γ4+F model UFboot from IQ-TREE, LG+Γ4+F model BP from RAxML, and CAT-GTR+Γ4 model Bayesian posterior probabilities from PhyloBayes-MPI. Filled circles represent maximal support (i.e. 100/100/1). Unlabelled branches received less than 50% UFboot support. Asterisks denote branches that were not recovered in inferred phylogeny for a given analysis.

Interestingly, removing rapidly evolving sites and rapidly-evolving taxa together revealed a broad ‘island’ of support for a malawimonads+metamonads clade (figure 4*d*). A single –FS/LB dataset from this island (23 000 fastest-evolving sites and 22 longest-branching taxa removed) was selected for detailed analysis. These analyses recovered a tree of eukaryotes mostly consistent with the initial phylogeny, but with different positions for malawimonads, metamonads and *Collodictyon* (figure 5). In this tree, malawimonads branched in a clade with Metamonada (in this case represented by *Trimastix* and *Paratrimastix*) that was quite strongly supported in the site-heterogeneous analysis (LG+C60+Γ4+F UFboot 92%), while bootstrap support under the LG+Γ4+F model was 81%, and posterior probability was low (0.7) in the Bayesian analysis (CAT-GTR+Γ4 model). This Malawimonadidae+Metamonada clade branched adjacent to Amorphea on the unrooted tree, while *Collodictyon* branched between this grouping and Discoba+Diaphoretickes (figure 5).

## 4. Discussion

The distant relationship between malawimonads and all other excavates inferred in most recent phylogenomic-scale analyses demands that the cell architecture of malawimonads be carefully re-evaluated, especially given their importance for understanding deep-level eukaryote evolution (e.g. [21,23]). If our re-examination of malawimonad ultrastructure had shown that the only cytoskeletal similarities between malawimonads and other excavates were those also shared by several other groups of eukaryotes [23], then the tension between morphology and typical phylogenomic results would disappear. Instead, our study shows the opposite, actually extending the known morphological similarity between malawimonads and (other) ‘typical excavates’.

The system of microtubular roots and supporting fibres that are general to ‘typical excavates’ (R1, splitting R2, singlet root, fibres ‘A’, ‘B’, ‘I’ and ‘C’; see [8]) are all present in *Gefionella okellyi,* as was proposed for *Malawimonas jakobiformis* [9]. Of these, the B fibre is most significant, since no unambiguous homologue of this structure has been positively identified outside of excavates (though see [41]). The confirmation here that the malawimonad B fibre is striated further supports its homology with the B fibres of other ‘typical excavates’ [8].

The two other best candidates for cytoskeletal synapomorphies for excavates are (i) the composite fibre, and (ii) the system of vanes on the posterior flagellum [8]. The composite fibre of *G. okellyi* is the first observed in a malawimonad. It is smaller than in most ‘typical excavates’, but is position-equivalent, and contains the standard arrangement of striated and dense components [10]. The absence of this fibre from the original description of *M. jakobiformis* [9] may be because that study focused on the cell's anterior, whereas the composite fibre is located posteriorly.

*Malawimonas jakobiformis* has vanes on the posterior flagellum, but is unusual in having a single ventral vane only [8,9]. By contrast, the pair of opposed vanes in *G. okellyi* conforms to the most common arrangement in metamonad ‘typical excavates’, which is inferred to be the ancestral state for Metamonada, based on mapping characters to molecular phylogenies [42]. Also, our documenting of striations on malawimonad vane lamellae further supports their homology with the lamellae of other ‘typical excavates’, which are similarly striated [8,43]. The possession of opposed vanes is shared by Malawimonadidae and Metamonada to the exclusion of Jakobida (the only ‘typical excavate’ group in Discoba), since jakobids have only a single dorsal vane [10].

Otherwise, the ultrastructure of *G. okellyi* underscores its identity as a malawimonad. The discoidal cristae, striated band and distal fibre connectives between the BBs, sizes of R1 and R2, and epipodium supported by part of R1 are all similar to *M. jakobiformis* [9]. The G fibre was not observed in *M. jakobiformis*, though this subtle feature would be easily overlooked. The conspicuous P fibre was also not recorded in *M. jakobiformis*, however, and provisionally distinguishes *Gefionella* from *Malawimonas*.

Meanwhile, our phylogenomic analyses demonstrate the weakness of the common inference that malawimonads are not related to any other excavates. It is well known that systematic errors such as long branch attraction (LBA) artefacts can result when the model of evolution does not sufficiently reflect the actual evolutionary process [44]. In some cases these errors can be overcome using more realistic models of sequence evolution, or excluding likely sources of phylogenetic ‘noise’, such as fast-evolving sites or long-branching taxa. On this basis, the relationship between malawimonads and *Collodictyon* recovered in our initial analysis (and several previous analyses [16,18,20]) is suspected to represent phylogenetic error. It is only weakly supported in our ML analysis using a site-heterogeneous mixture model (69% UFboot), and support under site-homogeneous substitution models rapidly decreases once several thousand fast-evolving sites are removed (figure 4*b*). In parallel, site-heterogeneous models support the conventional placement of Metamonada with Discoba only moderately (under ML with the LG+C60+Γ4+F model) or not at all (under Bayesian analysis with the CAT-GTR model), and the initially strong support for Metamonada+Discoba under simpler site-homogeneous models weakens as the noisiest data are excluded (fast-evolving sites in particular: electronic supplementary material, figure S6). The collapse in support for both groupings with exclusion of fast-evolving sites occurred while support for other similar-scale groupings remained very strong (exemplified by Opisthokonta in figure 4*b*, but equivalent for other clades). This indicates that the dissolution of support for Malawimonadidae+*Collodictyon* and Metamonada+Discoba is not due to a general loss of deep phylogenetic signal. Instead, we find support for a Malawimonadidae+Metamonada grouping in our ‘–FS/LB’ analyses, where fast-evolving sites and long branching taxa are both removed (figures 4*d* and 5). As pointed out by Derelle *et al*. [21], a malawimonad+metamonad relationship has also been observed in a few recent phylogenomic analyses, specifically some in which metamonads are represented solely by the shorter-branching species *Paratrimastix pyriformis* (formerly *Trimastix pyriformis*) [18,19,30]. Thus, from our various treatments, (i) a malawimonad+metamonad grouping received its strongest support from the slowest evolving sites, and (ii) we still recovered this grouping with better taxon sampling for malawimonads and short-branched metamonads than was available in previous work. These trends are consistent with the malawimonad+metamonad phylogenetic signal reflecting the true evolutionary history. Conversely, the initial topology, including a Metamonada+Discoba clade, may be affected by LBA. Prior to long-branch removal, Metamonada and Discoba each included some of the longest-branching taxa examined (electronic supplementary material, figure S5), even though the most divergent metamonad taxa (e.g. diplomonads) were excluded *a priori*. This could have resulted in metamonads being pulled toward discobids and away from malawimonads, the latter being one of the shortest-branching groups of eukaryotes.

A close relationship between malawimonads and metamonads would also be consistent with other non-phylogenomic data. As discussed above, there are considerable morphological similarities between malawimonads and metamonad ‘typical excavates’. At the ultrastructural level, they resemble each other more than either resemble any other group of eukaryotes, including other ‘typical excavates’ (i.e. jakobids), when the new information on flagellar vane organization is taken into account. (Malawimonads and metamonads also share the plesiomorphy of having an anterior R3 root, which is likely absent in all jakobids [43].) Further, some phylogenies inferred for one or a few slowly-evolving nucleus-encoded proteins place malawimonads with at least the shorter-branching metamonads (e.g. *Trimastix*, *Paratrimastix*), albeit usually with weak statistical support [11,13,14].

In summary, this study provides additional evidence that malawimonads are ‘typical excavates’, morphologically speaking, with their greatest similarity being to certain metamonads. Further, it highlights the weakness of the phylogenomic evidence separating malawimonads from all other excavates, and demonstrates a case where a moderately-well-supported malawimonad+metamonad grouping can be recovered in selected noise-filtered datasets. Together, the re-examined morphological and phylogenetic evidence imply that the predominant view of the evolutionary relationships among excavates (that malawimonads branch outside, and probably completely separately, from a robust Metamonada+Discoba clade [3–5]) is extremely insecure. Instead, the proposition that metamonads are more closely related to malawimonads than they are to Discoba is consistent with a greater range of evidence and analyses. Therefore, we caution that an Excavata grouping of Metamonada+Discoba (exclusively) should not be assumed in studies of the evolution of eukaryotes, such as inferring the history of major cellular systems from comparative genome data (e.g. [45–47]). In this view (and contrary to that with malawimonads branching separately from a Metamonada+Discoba clade), the strong morphological similarity between malawimonads and metamonads is not directly relevant for inferring the cytoskeleton organization in the last common ancestor of eukaryotes, since they are unlikely to branch on opposite sides of the root of eukaryotes [21].

The ‘malawimonad+metamonad signal’ may or may not reflect an exclusive sister-group relationship between these two taxa. Further testing is needed, especially using high-quality phylogenomic data from other hard-to-place eukaryote lineages (addressed in our ongoing research). In addition, the large disparities in branch lengths among Malawimonadidae, Metamonada and Discoba make it very challenging to resolve their relationships using phylogenomics. Denser and better-quality taxon sampling in phylogenomic datasets would be valuable, especially the addition of shorter-branching lineages of metamonads and discobids, or more malawimonad clades. Recent isolations of novel deep-branching discobids and metamonads [13,14,48] hint that many other important excavate lineages may indeed await discovery. Final resolution of these relationships will probably also require both sophisticated evolutionary models and identifying the most reliable data within the large amounts of sequence information now available.

## 5. Taxonomic summary and description

***Gefionella* Heiss, Ekelund and Simpson n. gen.**

**Diagnosis**. Malawimonad with two vanes on posterior flagellum, and conspicuous paired fibre (P) connecting basal body 2 and R2.

**Etymology**. Gefion-: Gefion (Gefjon, Gefjun) is a Norse goddess associated with ploughing; Gefionspringvandet, the largest public monument in Copenhagen, shows Gefion goading her oxen with a whip. -ella: Latin feminine diminutive. The name is appropriate for a small excavate(d) flagellate isolated from agricultural soil from Denmark. *Gefionella* is of feminine gender.

**Type species**. *Gefionella okellyi* Heiss, Ekelund and Simpson n. sp. (see below).

**Zoobank registration**. Described under the Zoological Code; Zoobank registration urn:lsid:zoobank.org:act:4CFB90BA-52A0-470F-9AD1-B2DF4BB59C09.

***Gefionella okellyi* Heiss, Ekelund and Simpson n. sp.**

**Diagnosis**. *Gefionella* species, cell body 4.4–7.2 µm long (not including occasional narrow posterior extensions).

**Type material**. The name-bearing type (an hapantotype) is a collection of fixed, dehydrated, resin-embedded cells of strain 249, deposited with the American Museum of Natural History, New York, as AMNH_IZC 00267131. This material also contains uncharacterized prokaryote prey.

**Type locality**. Agricultural soil, Foulum, Jutland, Denmark [56°29′47.8^″^ N 9°34′32.2^″^ E].

**Etymology**. Named after Charles J. O'Kelly, who pioneered ultrastructural and phylogenetic research into small ‘excavate’ flagellates, including malawimonads.

**Zoobank registration**. Described under the Zoological Code; Zoobank registration urn:lsid:zoobank.org:act:461B7908-6D63-4E79-8AE5-E7BAFAB28BA2.

## Supplementary Material

Supplementary Figure 1

## Supplementary Material

Supplementary Figure 2

## Supplementary Material

Supplementary Figure 3

## Supplementary Material

Supplementary Figure 4

## Supplementary Material

Supplementary Figure 5

## Supplementary Material

Supplementary Figure 6

## Supplementary Material

Supplementary Materials Description

## Data accessibility

The transcriptome data are accessed in GenBank SRA as Bioproject PRJNA400870. The unmasked single-gene alignments and masked concatenated phylogenomic datasets are accessed in Dryad (http://dx.doi.org/10.5061/dryad.9mv6d51) [49].
