# Supplementary figures and images for: Combined morphological and phylogenomic re-examination of malawimonads, a critical taxon for inferring the evolutionary history of eukaryotes
Source: R Soc Open Sci. 2018 Apr 4;5(4):171707. doi: 10.1098/rsos.171707 (PMC5936906; doi:10.1098/rsos.171707)

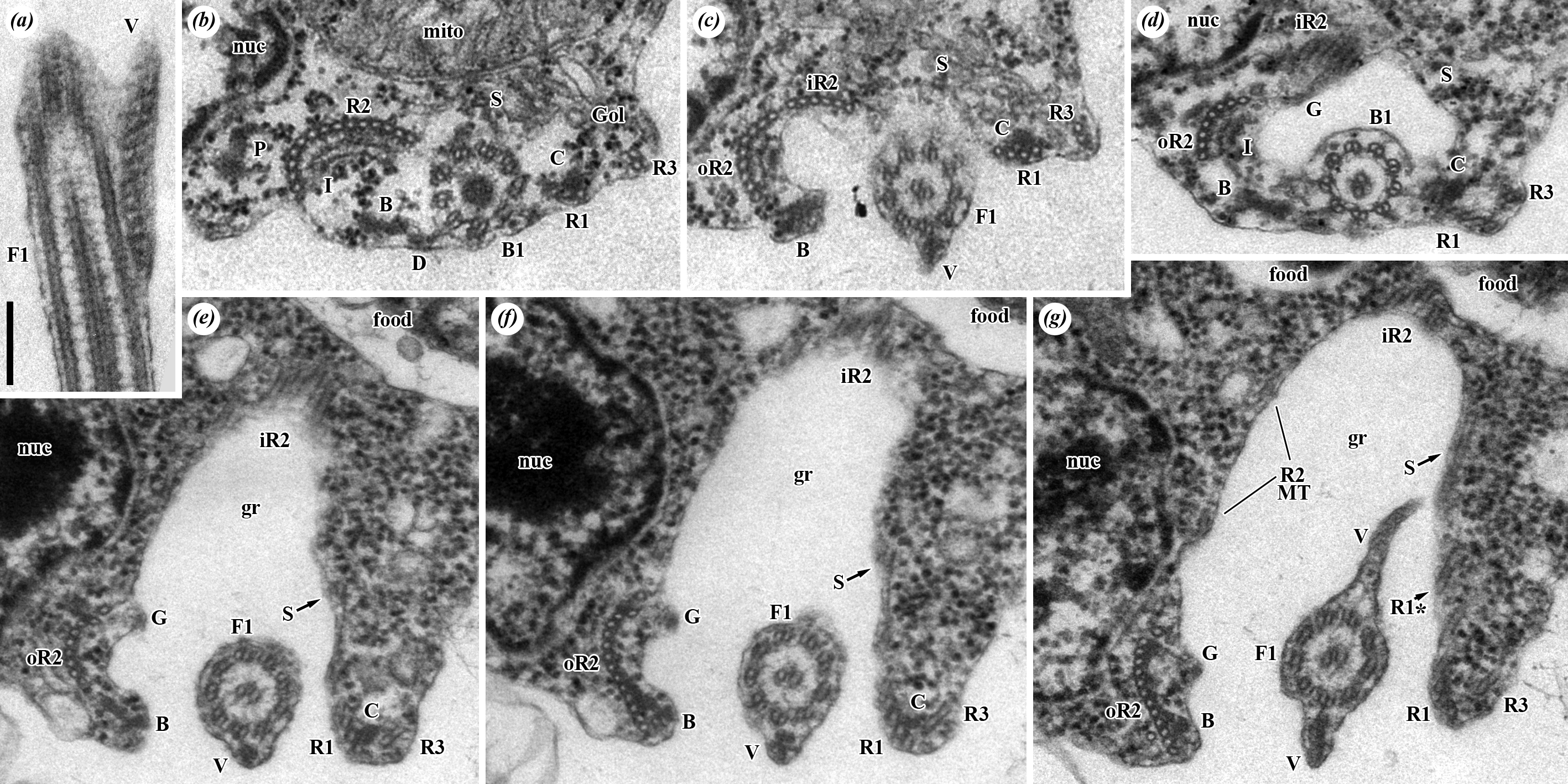

Supplement: Supplementary Figure 1 [file rsos171707supp1.tif]

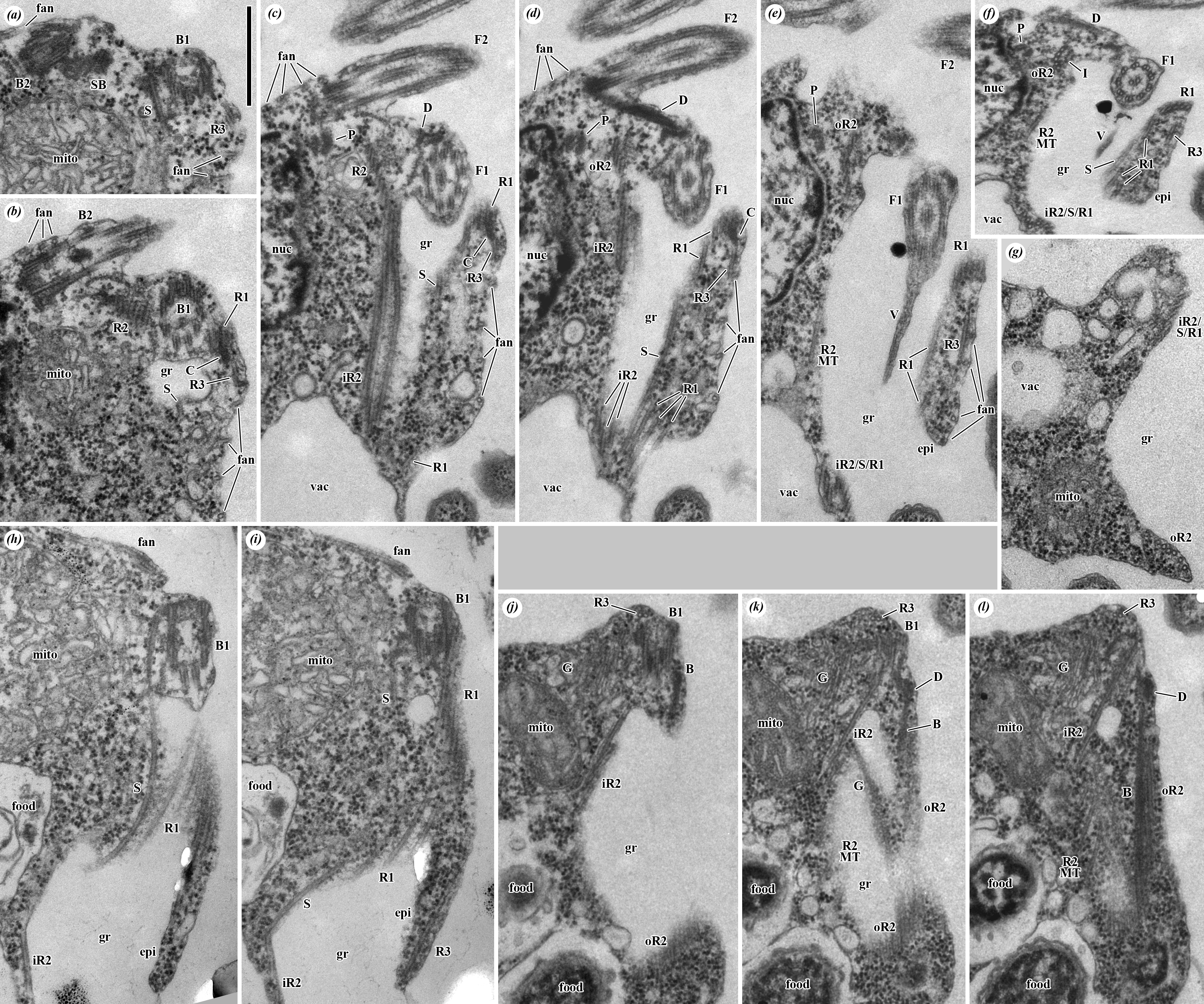

Supplement: Supplementary Figure 2 [file rsos171707supp2.tif]

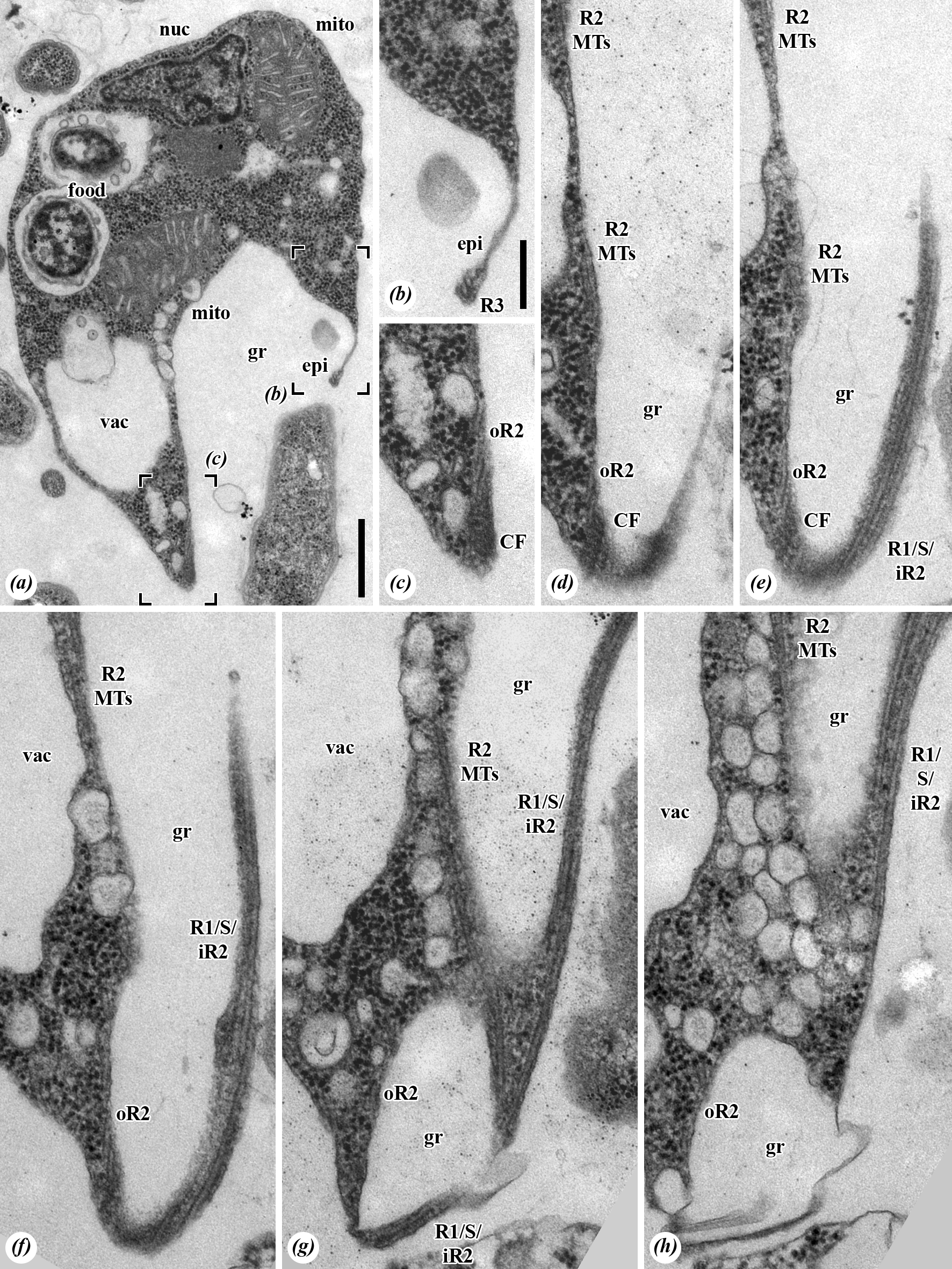

Supplement: Supplementary Figure 3 [file rsos171707supp3.tif]

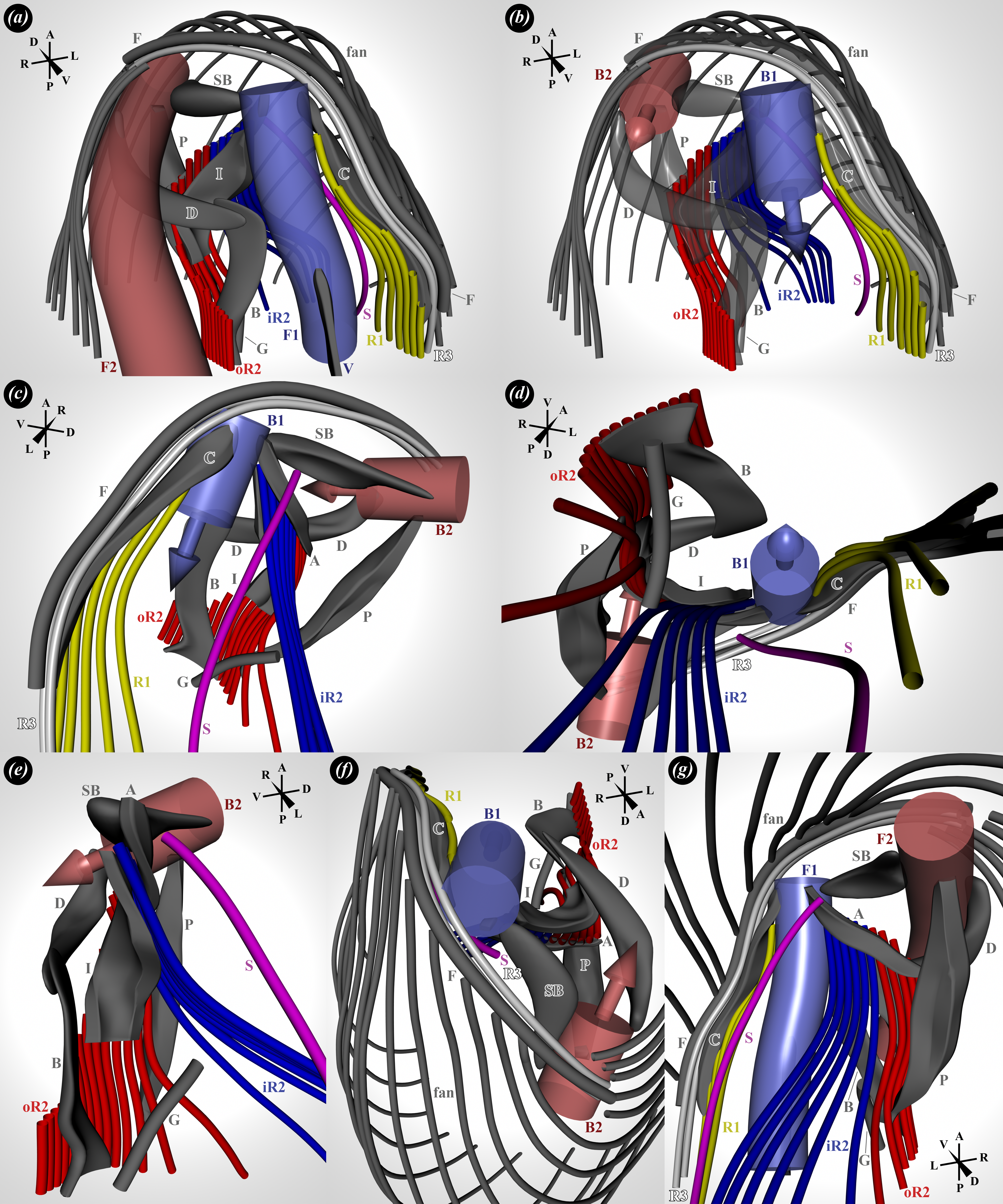

Supplement: Supplementary Figure 4 [file rsos171707supp4.tif]

# Support for Metamonada + Discoba

Taxa removed

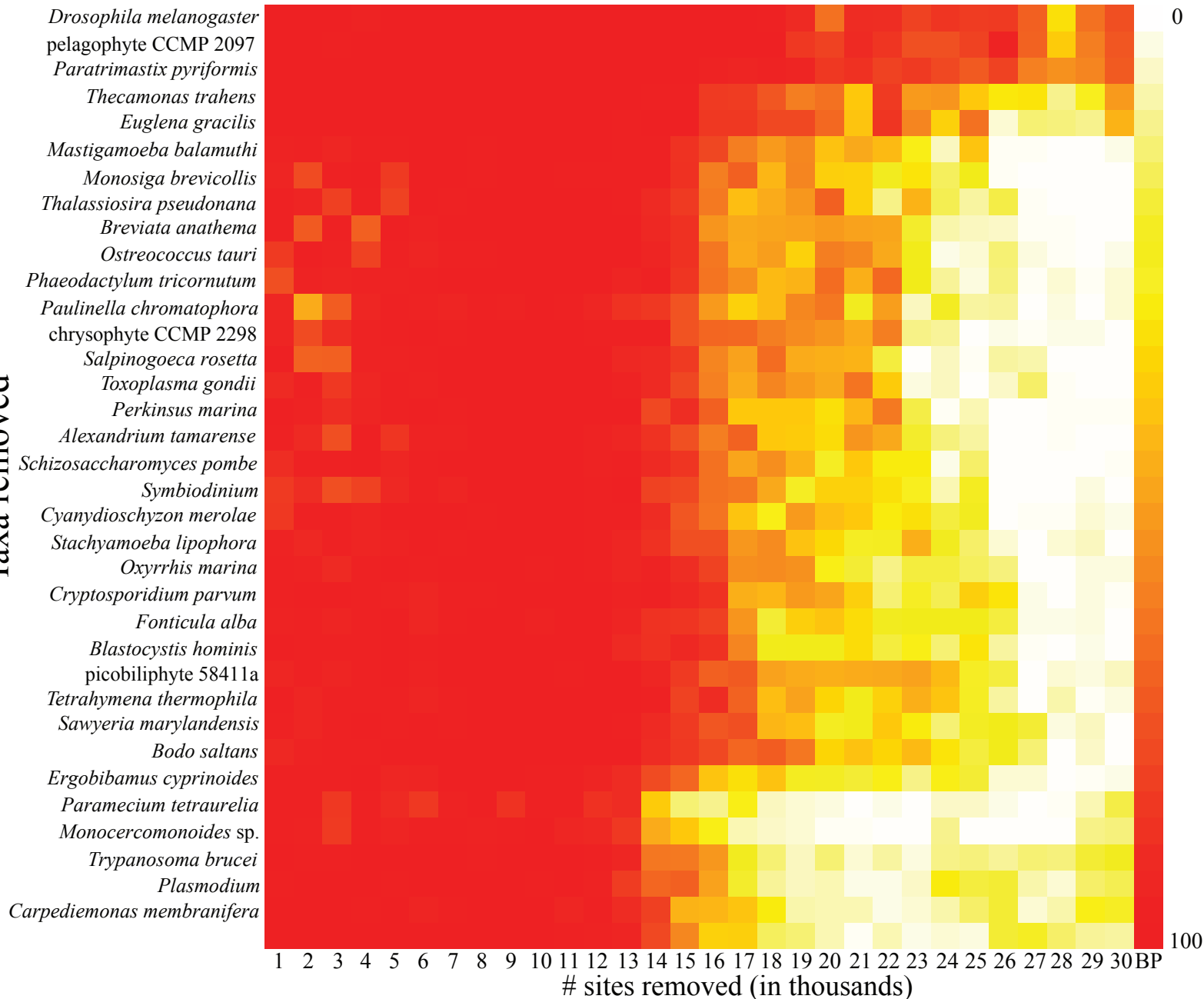

Supplement: Supplementary Figure 6 [file rsos171707supp6.pdf]
