# Supplementary material for: Combined morphological and phylogenomic re-examination of malawimonads, a critical taxon for inferring the evolutionary history of eukaryotes
Source: R Soc Open Sci. 2018 Apr 4;5(4):171707. doi: 10.1098/rsos.171707 (PMC5936906; doi:10.1098/rsos.171707)
Supplement: Supplementary Figure 5 [file rsos171707supp5.pdf]

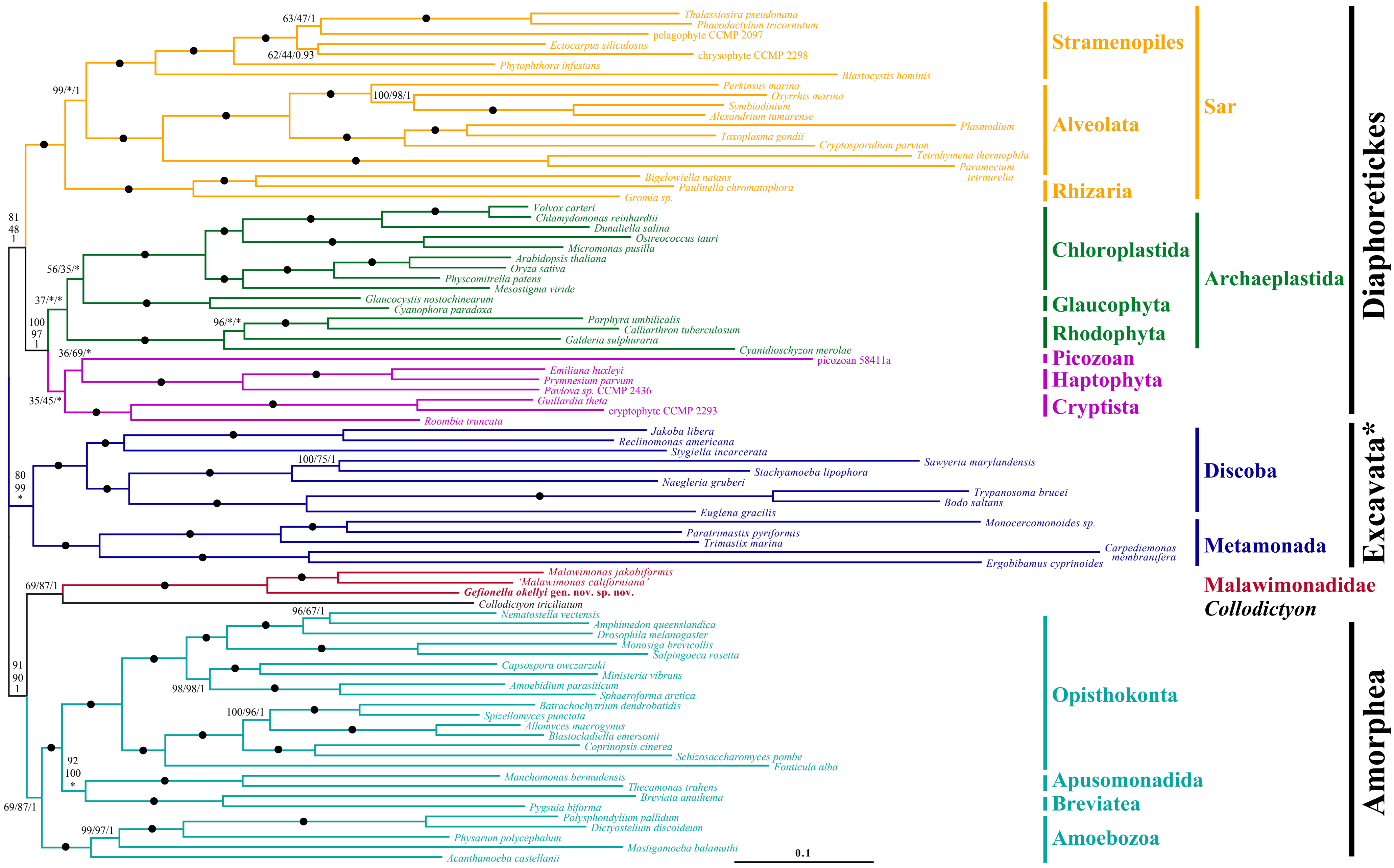
Thalassiosira pseudonana
Phaeodactylum tricornutum
pelagophyte CCMP 2097
Ectocarpus siliculosus
chrysophyte CCMP 2298
Phytophthora infestans
Blastocystis hominis
Perkinsus marina
Oxyrrhis marina
Symbiodinium
Alexandrium tamarense
Plasmodium
Toxoplasma gondii
Cryptosporidium parvum
Tetrahymena thermophila
Paramecium tetraurelia
Bigelowiella natans
Paulinella chromatophora
Gromia sp.
Volvox carteri
Chlamydomonas reinhardtii
Dunaliella salina
Ostreococcus tauri
Micromonas pusilla
Arabidopsis thaliana
Oryza sativa
Physcomitrella patens
Mesostigma viride
Glaucocystis nostochinearum
Cyanophora paradoxa
Porphyra umbilicalis
Calliarthron tuberculosum
Galderia sulphuraria
Cyanidioschyzon merolae
picozoan 58411a
Emiliana huxleyi
Prymnesium parvum
Pavlova sp. CCMP 2436
Guillardia theta
cryptophyte CCMP 2293
Roombia truncata
Jakoba libera
Reclinomonas americana
Stygiella incarcerata
Sawyeria marylandensis
Stachyamoeba lipophora
Naegleria gruberi
Trypanosoma brucei
Bodo saltans
Euglena gracilis
Monocercomonoides sp.
Paratrimastix pyriformis
Trimastix marina
Carpediemonas membranifera
Ergobibamus cyprinoides
Malawimonas jakobiformis
'Malawimonas californiana'
Gefionella okellyi gen. nov. sp. nov.
Collodictyon triciliatum
Nematostella vectensis
Amphimedon queenslandica
Drosophila melanogaster
Monosiga brevicollis
Salpingoeca rosetta
Capsospora owczarzaki
Ministeria vibrans
Amoebidium parasiticum
Sphaeroforma arctica
Batrachochytrium dendrobatidis
Spizellomyces punctata
Allomyces macrogynus
Blastocladiella emersonii
Coprinopsis cinerea
Schizosaccharomyces pombe
Fonticula alba
Manchomonas bermudensis
Thecamonas trahens
Breviata anathema
Pygsuia biforma
Polysphondylium pallidum
Dictyostelium discoideum
Physarum polycephalum
Mastigamoeba balamuthi
Acanthamoeba castellanii
63/47/1
62/44/0.93
99/*/1
100/98/1
81 48 1
56/35/*
37/*/*
100 97 1
96/*/*
36/69/*
35/45/*
100/75/1
80 99 *
69/87/1
96/67/1
98/98/1
100/96/1
91 90 1
92 100 *
69/87/1
99/97/1
0.1
Stramenopiles
Alveolata
Rhizaria
Sar
Chloroplastida
Glaucophyta
Rhodophyta
Archaeplastida
Picozoan
Haptophyta
Cryptista
Diaphoretickes
Discoba
Metamonada
Excavata*
Malawimonadidae
Collodictyon
Opisthokonta
Apusomonadida
Breviatea
Amoebozoa
Amorphea
