# Supplementary material for: Combined morphological and phylogenomic re-examination of malawimonads, a critical taxon for inferring the evolutionary history of eukaryotes
Source: R Soc Open Sci. 2018 Apr 4;5(4):171707. doi: 10.1098/rsos.171707 (PMC5936906; doi:10.1098/rsos.171707)
Supplement: Supplementary Materials Description [file rsos171707supp7.pdf]

Description of Supplementary Material for:

Combined morphological and phylogenomic re-examination of malawimonads, a critical taxon for inferring the evolutionary history of eukaryotes.

A. A. Heiss, M. Kolisko, F. Ekelund, M. W. Brown, A. J. Roger & A. G. B. Simpson

Supplementary Figure 1. Transmission electron microscopy (TEM) of *G. okellyi*: flagellar apparatus and architecture of anterior portion of groove. In all images (except a), the cell's left is on the right of image, and vice versa. (a) Posterior flagellum, showing terminating vane (V). Note striation of vane lamella. (b,c) Non-consecutive sections showing proximal part of posterior flagellar apparatus, highlighting origins and substructures of 'I' and 'B' fibres (supporting R2 microtubular root, especially outer 'oR2' portion) and 'C' fibre (supporting R1 microtubular root; note also close association with R3 microtubular root). (d–g) Non-consecutive series showing main structures supporting anterior portion of ventral groove. Note separation between iR2 and oR2, origin of 'G' fibre against iR2 (in d), termination of 'I' fibre (by e) and C fibre (by g); series also shows distinct origins of both flagellar vanes on posterior flagellum (F1). B1, basal body 1 (of posterior flagellum); C, 'C' fibre; D, dense fibre; F1, posterior flagellum; food, food vacuole; G, 'G' fibre; Gol, Golgi apparatus; gr, groove; I, 'I' fibre; iR2, inner portion of microtubular root 2; mito, mitochondrion; nuc, nucleus; oR2, outer portion of microtubular root 2; P, 'P' fibre; R1–R3, microtubular roots 1–3; R1*, microtubule from R1 illustrating beginning of 'fraying' of R1; R2MT, individual microtubules derived from R2; S, singlet microtubular root; V, flagellar vane. Scale bar: (a–g, in a) 200 nm.

Supplementary Figure 2. Transmission electron microscopy (TEM) of *G. okellyi*: groove architecture. (a–e) Non-consecutive series of oblique sections, focusing on left half of groove, showing microtubular support of epipodium, and showing formation of combined iR2/S/R1 microtubule group that supports left margin of posterior portion of groove. (f) High-tilt-angle image of section from same series as a–e (between d and e) detailing epipodium elements. (g)

Transverse section of groove in middle of cell (ventral is to right of image), showing microtubular support for groove margins. (h,i) Near-longitudinal sections focusing on left half of groove and epipodium (compare with a–f). (j–l) Near-longitudinal series showing anterior portion of right margin of groove. Note origins and paths of 'B' and 'G' fibres, as well as R2-derived microtubules (R2MT) between iR2 and oR2. B, 'B' fibre; B1, basal body 1 (of posterior flagellum); B2, basal body 2 (of anterior flagellum); C, 'C' fibre; D, dense fibre; epi, epipodium; F1, posterior flagellum; F2, anterior flagellum; fan, dorsal fan of microtubules; food, food vacuole; G, 'G' fibre; Gol, Golgi apparatus; gr, groove; I, 'I' fibre; iR2, inner portion of microtubular root 2; mito, mitochondrion; nuc, nucleus; oR2, outer portion of microtubular root 2; P, 'P' fibre; R1–R3, microtubular roots 1–3; R2MT, individual microtubules derived from R2; S, singlet microtubular root; SB, striated band; V, flagellar vane; vac, vacuole. Scale bar: (a–l, in a) 500 nm.

Supplementary Figure 3. Transmission electron microscopy (TEM) of *G. okellyi*: posterior portion of cell. In all images, cell's left is on right of image, and vice versa. (a) Oblique section through cell from left-anterior to right-posterior showing end of epipodium (on left margin of groove), and posterior portion of right margin of groove; note marks for the position of detail images b and c. (b) Detail of end of epipodium. (c) Detail of posterior portion of right groove margin, including composite fibre supporting remains of oR2. (d–h) Longitudinal sections through extreme posterior end of groove, showing composite fibre and oR2 (right margin) meeting R1/S/iR2 group (left margin). Note small number of R2-derived microtubules (R2MTs) cross obliquely leftward from right of groove, converging before posterior end of cell. CF, composite fibre; epi, epipodium; food, food vacuole; gr, groove; iR2, inner portion of microtubular root 2; mito, mitochondrion; nuc, nucleus; oR2, outer portion of microtubular root 2; P, 'P' fibre; R1, microtubular root 1; R3, microtubular root 3; R2MTs, individual microtubules derived from R2; S, singlet microtubular root; vac, vacuole. Scale bars: (a) 500 nm (b–h, in b) 200 nm.

Supplementary Figure 4. Alternative views of model of proximal portion of flagellar apparatus, rendered from 21-section series (See Fig. 3j); note compasses for orientation. Note full rendering of flagella in (a) and (g); in b–f basal bodies only are shown, with arrows starting at and showing direction of flagellar emergence. A, 'A' fibre; B, 'B' fibre; B1, basal body 1 (of posterior

flagellum); B2, basal body 2 (of anterior flagellum); C, ‘C’ fibre; D, dense fibre; F1, posterior flagellum; F2, anterior flagellum; fan, dorsal fan of microtubules; G, ‘G’ fibre; I, ‘I’ fibre; iR2, inner portion of microtubular root 2; oR2, outer portion of microtubular root 2; P, ‘P’ fibre; R1–R3, microtubular roots 1–3; S, singlet microtubular root; SB, striated band; V, vane (on F1).

Supplementary Figure 5. Full phylogenetic tree corresponding to summary tree shown in Fig. 4a. Analysis based on 159 genes, with all sites and 84 taxa included. Maximum likelihood (ML) tree shown was inferred under LG+C60+Γ4+F model of sequence evolution using IQTREE. Statistical support values are, in order: LG+C60+Γ4+F model ultrafast bootstrap approximation (UFboot) from IQTREE, LG+Γ4+F model bootstrap support (BP) from RAxML, and CAT-GTR+Γ4 model Bayesian posterior probabilities (from two converged chains) in PhyloBayes-MPI. Filled circles represent maximal support (i.e., 100/100/1.0). Asterisks denote branches that were not recovered in inferred phylogeny for a given analysis. Taxa in pink represent ‘other Diaphoretickes’, not a hypothesized major group. ‘Excavata’ is labelled with asterisk to signify that this clade does not include the ‘excavate taxon’ Malawimonadidae, and to also flag the contested nature of this clade (compare to figure 5).

Supplementary Figure 6. Heat map showing RAxML rapid bootstrap support for clade of Discoba and Metamonada with removal of fast-evolving sites (X-axis, in thousands) and fast-evolving taxa (Y-axis). Pure white denotes 0% BP and pure red denotes 100% BP (right-most column — “BP” — demonstrates color scale). Analyses performed using LG+CAT+F model and rapid bootstrapping in RAxML.
